# Factors associated with longer wait times, admission and reattendances in older patients attending emergency departments: an analysis of linked healthcare data

**DOI:** 10.1136/emermed-2022-212303

**Published:** 2023-01-17

**Authors:** Laia Maynou, Andrew Street, Christopher Burton, Suzanne M Mason, Tony Stone, Graham Martin, James van Oppen, Simon Conroy

**Affiliations:** 1 Department of Econometrics, Statistics and Applied Economics, Universitat de Barcelona, Barcelona, Spain; 2 Department of Health Policy, The London School of Economics and Political Science, London, UK; 3 Center for Research in Health and Economics (CRES), Universitat Pompeu Fabra, Barcelona, Spain; 4 Centre for Urgent and Emergency Care Research, School of Health and Related Research, The University of Sheffield, Sheffield, UK; 5 THIS Institute, University of Cambridge, Cambridge, UK; 6 Department of Health Sciences, University of Leicester, Leicester, UK; 7 Medical Research Council Unit for Lifelong Health and Ageing, University College London, London, UK

**Keywords:** emergency department, emergency ambulance systems, emergency care systems, geriatrics

## Abstract

**Background and objective:**

Care for older patients in the ED is an increasingly important issue with the ageing society. To better assess the quality of care in this patient group, we assessed predictors for three outcomes related to ED care: being seen and discharged within 4 hours of ED arrival; being admitted from ED to hospital and reattending the ED within 30 days. We also used these outcomes to identify better-performing EDs.

**Methods:**

The CUREd Research Database was used for a retrospective observational study of all 1 039 251 attendances by 368 754 patients aged 75+ years in 18 type 1 EDs in the Yorkshire and the Humber region of England between April 2012 and March 2017. We estimated multilevel logit models, accounting for patients’ characteristics and contact with emergency services prior to ED arrival, time variables and the ED itself.

**Results:**

Patients in the oldest category (95+ years vs 75–80 years) were more likely to have a long ED wait (OR=1.13 (95% CI=1.10 to 1.15)), hospital admission (OR=1.26 (95% CI=1.23 to 1.29)) and ED reattendance (OR=1.09 (95% CI=1.06 to 1.12)). Those who had previously attended (3+ vs 0 previous attendances) were more likely to have long wait (OR=1.07 (95% CI=1.06 to 1.08)), hospital admission (OR=1.10 (95% CI=1.09 to 1.12)) and ED attendance (OR=3.13 (95% CI=3.09 to 3.17)). Those who attended out of hours (vs not out of hours) were more likely to have a long ED wait (OR=1.33 (95% CI=1.32 to 1.34)), be admitted to hospital (OR=1.19 (95% CI=1.18 to 1.21)) and have ED reattendance (OR=1.07 (95% CI=1.05 to 1.08)). Those living in less deprived decile (vs most deprived decile) were less likely to have any of these three outcomes: OR=0.93 (95% CI=0.92 to 0.95), 0.92 (95% CI=0.90 to 0.94), 0.86 (95% CI=0.84 to 0.88). These characteristics were not strongly associated with long waits for those who arrived by ambulance. Emergency call handler designation was the strongest predictor of long ED waits and hospital admission: compared with those who did not arrive by ambulance; ORs for these outcomes were 1.18 (95% CI=1.16 to 1.20) and 1.85 (95% CI=1.81 to 1.89) for those designated less urgent; 1.37 (95% CI=1.33 to 1.40) and 2.13 (95% CI=2.07 to 2.18) for urgent attendees; 1.26 (95% CI=1.23 to 1.28) and 2.40 (95% CI=2.36 to 2.45) for emergency attendees; and 1.37 (95% CI=1.28 to 1.45) and 2.42 (95% CI=2.26 to 2.59) for those with life-threatening conditions. We identified two EDs whose patients were less likely to have a long ED, hospital admission or ED reattendance than other EDs in the region.

**Conclusions:**

Age, previous attendance and attending out of hours were all associated with an increased likelihood of exceeding 4 hours in the ED, hospital admission and reattendance among patients over 75 years. These differences were less pronounced among those arriving by ambulance. Emergency call handler designation could be used to identify those at the highest risk of long ED waits, hospital admission and ED reattendance.

**WHAT IS ALREADY KNOWN ON THIS TOPIC**

- Policymakers in England have attempted to improve the performance of EDs mainly by setting and monitoring targets for how quickly patients are managed. Other outcomes such as admission and reattendance have not been a focus of these efforts. Moreover, while it is known that longer ED waits are related to patient and ED characteristics, studies have not accounted for use of emergency calls and ambulances prior to attendance.

**WHAT THIS STUDY ADDS**

- Using readily available system-level data, we analysed a triple metric of ED service outcomes, namely long ED waits, hospital admission and ED reattendance among 18 EDs in Yorkshire and the Humber. We control for a rich set of variables including patient characteristics, calls to emergency services and use of ambulance services prior to ED attendance, the time and day of attendance, and the size and staffing of the ED.
- For those conveyed to the ED by ambulance, we identified a strong association between call handler designation of urgency with outcomes.
- We identified 2 of 18 EDs whose older patients were less likely to wait more than 4 hours, to be admitted to hospital or to reattend within 30 days.

**HOW THIS STUDY MIGHT AFFECT RESEARCH, PRACTICE OR POLICY**

- Call handler designation of urgency could be easily used to identify those at the highest risk of these outcomes upon arrival at ED.
- The triple metric could be used as a measure of quality in ED care for older adults.

## Introduction

A growing evidence base for holistic care for older patients in emergency care settings demonstrates improved outcomes from person-centred, multidisciplinary, boundary-spanning care pathways.^1–6^ Despite this, policy in many countries has focused on ED waiting times, such as the 4-hour standard in England which has not been achieved for some years,^7^ overlookin g outcomes such as whether patients were admitted to hospital and reattended the ED. Appraisal of urgent care provision should also take account of the wider determinant of outcomes, such as patient, pathway and provider characteristics. The ED, although important, is but one part of a wider urgent care pathway that includes services that patients used prior to attending the ED, such as their emergency telephone calls and use of ambulance services. To determine the impact a specific ED can have on outcomes requires having an available large set of variables in a single database to allow us to control for patient sociodemographic and clinical characteristics, times and days of attendance, previous use of urgent care services, location, size and staffing of hospitals.

The aims of this paper were to identify patient and urgent care pathway characteristics that predict whether patients aged 75+ years were seen and discharged within 4 hours of ED arrival, were admitted from ED to hospital and reattended the ED within 30 days; and to identify better-performing EDs with respect to these outcomes.

## Methods

In this retrospective observational study, we analysed linked routine healthcare data for older patients (75+ years) who attended type 1 EDs between April 2012 and March 2017 in the Yorkshire and the Humber (Y&H) region of the UK (total population 5.5 million). Emergency care in the region over the period was provided by one ambulance service (Yorkshire Ambulance Service) with a single emergency phone number (999), an NHS telephone triage service (NHS111) and 18 type 1 EDs (24-hour consultant-led services with resuscitation facilities) across 13 acute hospital trusts. We extracted data from the CUREd Research Database (https://www.sheffield.ac.uk/scharr/research/centres/cure/projects) which collates routine NHS data from NHS111, the Yorkshire Ambulance Service Computer Aided Dispatch Data and the ED Patient Administration Systems. The database makes it possible to track each attendance from the person’s initial emergency contact, through transit to the ED, to transfer from the ED.^8^

The dataset contains data on 368 754 patients aged 75+ years who attended any of the 18 type 1 EDs in the Y&H region on one or more occasions between April 2012 and March 2017. Many patients attended the ED on multiple occasions, generating 1 039 251 separate attendances, with 1% of patients attending more than 12 times over the full period. When there were multiple attendances on the same day, we analysed only the last attendance of the day. This meant 8988 out of 1 039 251 (0.86%) attendances were excluded from analyses.

Taking ED attendances as the unit of analysis, we analysed three patient outcomes: whether they were seen and discharged from the ED within 4 hours of arrival; whether they were admitted from ED to hospital; and whether they reattended the ED within 30 days of discharge either from the ED or hospital. Each outcome was analysed using a multilevel logit model, with the effect of each explanatory variable expressed in ORs.

The analyses controlled for the patients’ age, sex, socioeconomic status using deciles of the Index of Multiple Deprivation (IMD),^9^ number of emergency admissions in the past year, whether patients were care home residents, and for the estimated travel time by road between the patient’s residence and the hospital.^10^

We included variables capturing the patient’s emergency and urgent care journey prior to ED attendance, namely: the number and length of the individual’s emergency (NHS111 and 999) calls; and, for those conveyed to the ED by ambulance, the urgency with which calls were accorded priority by call handlers using algorithms; time of the ambulance on scene (arrival to departure); and time taken between calling the ambulance and arrival at the ED. Those who did not make an emergency telephone call or use ambulance services were included in the analyses, but assigned values of zero for these variables act as reference categories in the main analyses.

We accounted for out-of-hours ED attendances occurring on weekends, public holidays and weekdays from 18:30 to 08:00. We controlled for daily, seasonal and annual trends by including day, month and year variables (estimates for these variables are suppressed in the tables and figures in the main paper but reported in the online supplemental material 1appendix Table A1 and Figure A1). ED size was measured using the number of attendances (in 1000s) during the year that the patient attended, and differences in staffing ratios across EDs were captured by the number of attendances divided by the number of senior doctors (accredited emergency physicians). The analysis of reattendance was conditioned on the patient surviving the ED or hospital admission.

10.1136/emermed-2022-212303.supp1 Supplementary data

A random effect was included for each ED site and for each patient, recognising that they might have multiple attendances, perhaps to different EDs. The random effect for each ED site is interpreted as a measure of relative performance, capturing the influence of the ED on each outcome after controlling for all the other explanatory factors.^11^

The main analyses were conducted for the full set of observations. As a robustness check, we also controlled for the percentage of available overnight hospital beds that are occupied using quarterly data. This variable was not part of the main analyses because, when included, the model estimating the probability of hospital admission failed to converge unless the year dummies were omitted. Results are reported in the online supplemental material 1 appendix (Table A2 and Figure A2).

We also conducted subgroup analyses for those who arrived at the ED by ambulance and for those who came by other means. In our analysis of whether patients waited more than 4 hours in the ED, we performed a separate subgroup analysis just for those who were not subsequently admitted to hospital.

Statistical analysis was conducted using Stata V.15 (College Station, Texas, USA).

### Patient and public involvement

Patients, carers and public representatives from the Leicester, Leicestershire, and Rutland Older People’s Patient and Public Involvement forum were involved in this study from its inception. Members of the forum were part of the project management team and involved in reviewing the emerging findings.

## Results

### Characteristics of study subjects

**Figure 1:**
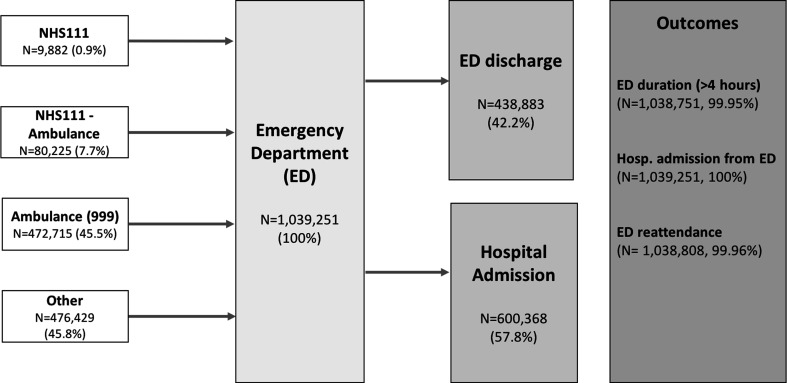
Emergency care pathways, 2012–2017 (type 1–18 sites). N for outcomes indicates those without missing data. Source: CUREd dataset.

The dataset comprised a total of 1 039 251 ED attendances made by 368 754 patients aged 75+ years between April 2012 and March 2017. Figure 1 summarises the possible routes to the ED and table 1 reports summary statistics across all observations with non-missing values for each of the outcome and explanatory variables. Observations with missing data for any variable were dropped, resulting in samples of 990 172 (95.28%), 990 645 (95.32%) and 990 229 (95.28%) for the analyses of long waits, hospital admission and ED reattendance, respectively.

**Table 1:** Descriptive statistics: outcomes and covariates (2012–2017)

Variables	N	n	%
ED outcomes			
ED duration (>4 hours)	1 038 751	293 102	28.22
Hospital admission from ED	1 039 251	600 368	57.77
ED reattendance within 30 days	1 038 808	212 050	20.41
Patient characteristics			
Age (median, IQR)	1 039 251	83	9
Age 75–80	1 039 251	302 234	29.08
Age 80–85	1 039 251	305 512	29.40
Age 85–90	1 039 251	250 855	24.14
Age 90–95	1 039 251	138 961	13.37
Age 95+	1 039 251	41 689	4.01
Sex (=1 male)	1 039 217	426 084	41.00
IMD1—most deprived	1 034 589	184 685	17.85
IMD2	1 034 589	103 025	9.96
IMD3	1 034 589	110 259	10.66
IMD4	1 034 589	87 630	8.47
IMD5	1 034 589	91 991	8.89
IMD6	1 034 589	103 182	9.97
IMD7	1 034 589	103 367	9.99
IMD8	1 034 589	89 697	8.67
IMD9	1 034 589	89 424	8.64
IMD10—least deprived	1 034 589	71 329	6.89
Number of previous ED attendances (1-year window) (median, IQR)	995 300	1	2
Number of previous admissions (1-year window)=0	995 300	451 990	45.41
Number of previous admissions (1-year window)=1	995 300	239 172	24.03
Number of previous admissions (1-year window)=2	995 300	127 665	12.83
Number of previous admissions (1-year window)=3+	995 300	176 473	17.73
Care home (=1 yes)	1 035 437	176 613	17.06
Road travel distance (min) (LSOA to hospital) (median, IQR)	1 034 814	10	10
Death in ED or hospital	1 039 251	52 705	5.07
Pathway variables			
Length NHS111 call (min)* (median, IQR)	90 107	10.22	8.90
Calls to NHS111 per day (>1)*	90 107	5774	6.41
Ambulance calls per day (>1)*	552 940	24 595	4.45
CH designation—less urgent	552 940	264 694	47.87
CH designation—urgent	552 940	96 199	17.40
CH designation—emergency	552 940	187 033	33.83
CH designation—life-threatening	552 940	5014	0.91
Time ambulance on scene (min)* (median, IQR)	538 087	36.1	21.65
Time from ambulance call to ED arrival (min)*† (median, IQR)	552 940	71.15	38.3
Site variables			
Site size in 1000 (financial year) (median, IQR)	1 039 251	11.92	6.92
Number of attendances per consultant (financial year) (median, IQR)	1 039 251	1388.50	967.03
Occupancy rate per trust (quarterly) (median, IQR)	1 039 251	84.45	7.29
Time variables			
Out of hours in ED (=1 yes)	1 039 251	562 545	54.13
Monday	1 039 251	156 211	15.03
Tuesday	1 039 251	146 475	14.09
Wednesday	1 039 251	145 676	14.02
Thursday	1 039 251	145 886	14.04
Friday	1 039 251	148 965	14.33
Saturday	1 039 251	149 051	14.34
Sunday	1 039 251	146 987	14.14
Bank holiday (=1 yes)	1 039 251	29 706	2.86
January	1 039 251	91 581	8.81
February	1 039 251	81 889	7.88
March	1 039 251	89 752	8.64
April	1 039 251	84 017	8.08
May	1 039 251	87 292	8.40
June	1 039 251	83 788	8.06
July	1 039 251	85 871	8.26
August	1 039 251	85 107	8.19
September	1 039 251	82 874	7.97
October	1 039 251	86 338	8.31
November	1 039 251	84 768	8.16
December	1 039 251	95 974	9.23

*In the regressions, we assign 0 min to the rest of the observations.

†Ambulance on scene has missing values in the departure time.

%, percentage of observations with characteristic; CH, call handler; IMD, Index of Multiple Deprivation; LSOA, Lower Super Output Area; n, observations with characteristic; N, total observations.

Of the total attendances, 90 107 (8.7%) first phoned NHS111, 6.4% making more than one call that same day and the average call lasting 15 min (SD 14), and were either advised to go directly to the ED (9882, 1.0%) or were taken by ambulance (80 225, 7.7%). A further 472 715 (45.5%) made a 999 call to the ambulance service and were transferred to the ED by ambulance. A total of 4.8% of these called 999 more than once that same day. Of all the 552 940 conveyances by ambulance, 47.9% were designated less urgent (category 4) by the call handlers, 17.4% urgent (category 3), 33.8% emergency (category 2) and 0.9% life-threatening (category 1).^12^ The average time between the call for an ambulance and arrival at the ED was 80 min (SD 40), with the average time on scene being 40 min (SD 19).

The remaining 476 429 (45.8%) attendances were self-presentations and involved no prior phone contact with NHS111 or 999 and no ambulance journey.

The mean age of those attending was 83.6 years (SD 5.9), 41% were male, 17.9% lived in areas in the most deprived decile (IMD1) with 7% in the least deprived decile (IMD10). A total of 45.4% had not attended an ED in the preceding 12 months but 17.7% had attended three times or more; 17.1% of attendances were by care home residents and the average travel time was 13 min (SD 9) from the patient’s place of residence to the ED. Fifty-four per cent of ED attendances were out of hours; there was a little difference across days of the week when patients attended, but higher proportions attended in December, January and February than other months.

A total of 293 102 (28.2%) attendances lasted more than 4 hours. A total of 4722 (0.5%) attendees died in the ED and 434 161 (41.8%) were cared for within and then discharged from the ED, while the remaining 600 368 (57.8%) were admitted to hospital for further treatment, of whom 48 017 (8%) died. A total of 212 050 (20.4%) reattended within 30 days of discharge.

### Factors influencing ED outcomes

**Figure 2:**
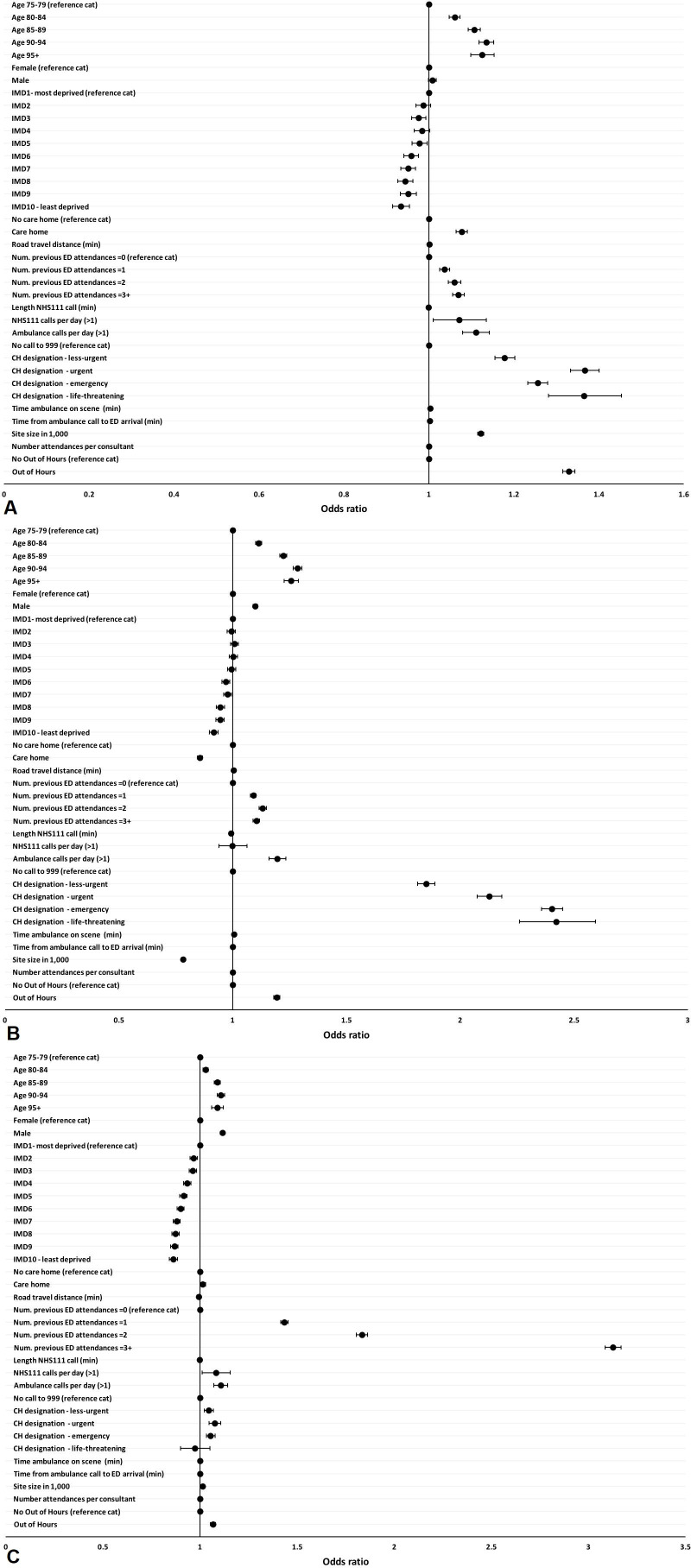
ED outcome estimates (2012–2017). (A) ED duration (>4 hours); (B) hospital admission from ED; (C) ED reattendance within 30 days. CH, call handler; IMD, Index of Multiple Deprivation.

Figure 2 and table 2 present the results from the analyses of the three ED outcomes. Compared with those aged 75–79 years, patients aged 95+ years were more likely to stay more than 4 hours in the ED (OR=1.125, 95% CI=1.098 to 1.153), to be admitted to hospital (OR=1.257, 95% CI=1.226 to 1.288) and to reattend the ED within 30 days (OR=1.089, 95% CI=1.059 to 1.119). Men were more likely to be admitted to hospital (OR=1.099, 95% CI=1.089 to 1.109) and had a higher likelihood of ED reattendance (OR=1.116, 95% CI=1.005 to 1.128). Compared with those living in areas in the most deprived decile, those from the least deprived decile (IMD10) were less likely to spend more than 4 hours in the ED (OR=0.934, 95% CI=0.915 to 0.954), to be admitted to hospital (OR=0.917, 95% CI=0.897 to 0.936) or to reattend (OR=0.863, 95% CI=0.842 to 0.884).

**Table 2:** Multilevel model—ED outcome results: ED duration, hospital admission from ED and ED reattendance within 30 days (2012–2017)

Dep var	(1)	(2)	(3)
	ED duration (>4 hours)	Hospital admission from ED	30-day reattendance
	Logit—OR	Logit—OR	Logit—OR
Age 75–80 (reference cat)	1	1	1
Age 80–85	1.061*** (1.048 to 1.073)	1.115*** (1.102 to 1.128)	1.028*** (1.015 to 1.042)
Age 85–90	1.107*** (1.093 to 1.121)	1.222*** (1.207 to 1.237)	1.088*** (1.073 to 1.104)
Age 90–95	1.135*** (1.118 to 1.153)	1.285*** (1.266 to 1.305)	1.107*** (1.089 to 1.126)
Age 95+	1.125*** (1.098 to 1.153)	1.257*** (1.226 to 1.288)	1.089*** (1.059 to 1.119)
Female (reference cat)	1	1	1
Male	1.008* (0.999 to 1.017)	1.099*** (1.089 to 1.109)	1.116***(1.105 to 1.128)
IMD1—most deprived (reference cat)	1	1	1
IMD2	0.986 (0.969 to 1.004)	0.994 (0.977 to 1.012)	0.967*** (0.949 to 0.986)
IMD3	0.976*** (0.959 to 0.993)	1.007 (0.990 to 1.025)	0.963*** (0.945 to 0.982)
IMD4	0.984* (0.965 to 1.002)	1.003 (0.984 to 1.022)	0.934*** (0.914 to 0.954)
IMD5	0.978** (0.960 to 0.996)	0.995 (0.977 to 1.014)	0.915*** (0.896 to 0.934)
IMD6	0.958*** (0.941 to 0.975)	0.970*** (0.952 to 0.987)	0.900*** (0.882 to 0.919)
IMD7	0.951*** (0.934 to 0.969)	0.978** (0.960 to 0.995)	0.880*** (0.862 to 0.898)
IMD8	0.944*** (0.926 to 0.962)	0.946*** (0.929 to 0.964)	0.874*** (0.856 to 0.893)
IMD9	0.951*** (0.933 to 0.970)	0.945*** (0.927 to 0.963)	0.868*** (0.849 to 0.887)
IMD10—least deprived	0.934*** (0.915 to 0.954)	0.917*** (0.897 to 0.936)	0.863*** (0.842 to 0.884)
No care home (reference cat)	1	1	1
Care home	1.077*** (1.064 to 1.090)	0.856*** (0.846 to 0.867)	1.014** (1.001 to 1.028)
Road travel distance (min)	1.001*** (1.001 to 1.002)	1.005*** (1.005 to 1.006)	0.993*** (0.992 to 0.994)
Number of previous ED attendances=0 (reference cat)	1	1	1
Number of previous ED attendances=1	1.037*** (1.025 to 1.049)	1.091*** (1.079 to 1.104)	1.434*** (1.415 to 1.453)
Number of previous ED attendances=2	1.060*** (1.045 to 1.076)	1.132*** (1.116 to 1.148)	1.834*** (1.806 to 1.863)
Number of previous ED attendances=3+	1.069*** (1.056 to 1.083)	1.104*** (1.090 to 1.118)	3.126*** (3.085 to 3.169)
Length NHS111 call (min)	0.999*** (0.998 to 0.999)	0.992*** (0.992 to 0.993)	0.999*** (0.998 to 0.999)
NHS111 calls per day (≤1) (reference cat)	1	1	1
NHS111 calls per day (>1)	1.071** (1.010 to 1.135)	0.999 (0.939 to 1.064)	1.081** (1.012 to 1.155)
Ambulance calls per day (≤1) (reference cat)	1	1	1
Ambulance calls per day (>1)	1.110*** (1.079 to 1.142)	1.196*** (1.160 to 1.234)	1.107*** (1.071 to 1.143)
No call to 999 (reference cat)	1	1	1
CH designation—less urgent	1.178*** (1.155 to 1.202)	1.850*** (1.813 to 1.888)	1.045*** (1.022 to 1.069)
CH designation—urgent	1.367*** (1.334 to 1.400)	2.128*** (2.074 to 2.183)	1.076*** (1.046 to 1.107)
CH designation—emergency	1.256*** (1.233 to 1.279)	2.403*** (2.357 to 2.449)	1.054*** (1.033 to 1.077)
CH designation—life-threatening	1.365*** (1.281 to 1.453)	2.422*** (2.261 to 2.594)	0.972 (0.899 to 1.052)
Time ambulance on scene (min)	1.003*** (1.002 to 1.003)	1.006*** (1.005 to 1.006)	1.001** (1.0001 to 1.001)
Time from ambulance call to ED arrival (min)	1.002*** (1.001 to 1.002)	0.999*** (0.999 to 0.999)	1.0004*** (1.0002 to 1.001)
No out of hours (reference cat)	1		
Out of hours (=1 yes)	1.329*** (1.315 to 1.344)	1.194*** (1.181 to 1.208)	1.067*** (1.054 to 1.081)
Site size in 1000	1.122*** (1.115 to 1.129)	0.781*** (0.777 to 0.786)	1.014*** (1.008 to 1.020)
Number of attendances per consultant	0.999*** (0.999 to 0.999)	1.0001*** (1.0001 to 1.0001)	1.000* (1.000 to 1.00004)
Var(j)	0.821*** (0.281 to 1.362)	2.260*** (0.784 to 3.736)	0.009*** (0.003 to 0.014)
N	990 172	990 645	990 229
Sites	18	18	18

Significance levels: ***p<0.01, **p<0.05, *p<0.1. ORs reported and 95% CIs in parentheses. Variation in the random effects across sites is captured by Var(j) reported as mean of the variance with the 95% CIs in parentheses. Model 3 (30-day reattendance) includes death as a covariate.

CH, call handler; IMD, Index of Multiple Deprivation; N, total observations.

Care home residents were more likely to spend more than 4 hours in the ED (OR=1.077, 95% CI=1.064 to 1.090) and less likely to be admitted to hospital (OR=0.856, 95% CI=0.846 to 0.867). Patients living closer to hospital were more likely to be admitted to hospital (OR=1.005, 95% CI=1.005 to 1.006) and were less likely to reattend (OR=0.993, 95% CI=0.992 to 0.994). Previous attendances were significantly associated with all outcomes, particularly for frequent attendees. Compared with those who had not previously attended the ED in the past year, those who had attended three or more times were more likely to spend more than 4 hours in the ED (OR=1.069, 95% CI=1.056 to 1.083), to be admitted to hospital (OR=1.104, 95% CI=1.090 to 1.118) and to reattend the ED within 30 days (OR=3.126, 95% CI=3.085 to 3.169).

Patients were more likely to spend more than 4 hours in the ED or to reattend if they made more than one NHS111 or 999 call on the day of ED attendance. For those conveyed by ambulance, the call handler designation of urgency was very strongly associated with all three outcomes. Compared with those who made their own way to the ED, the probability of waiting more than 4 hours was higher for those designated as less urgent (OR=1.178, 95% CI=1.155 to 1.202), urgent (OR=1.367, 95% CI=1.334 to 1.400), emergency (OR=1.256, 95% CI=1.233 to 1.279) and with a life-threatening condition (OR=1.365, 95% CI=1.281 to 1.453). There is also a clear gradient across urgency categories in the likelihood of hospital admission, increasing from OR=1.850 (95% CI=1.813 to 1.888) for those designated less urgent to OR=2.422 (95% CI=2.261 to 2.594) for those with life-threatening conditions. This pattern is not evident, and the effects are smaller or insignificant, when considering reattendance. The longer the ambulance was on the scene and the longer the journey to the ED, the higher the likelihood that the patient stayed more than 4 hours in the ED.

Patients who attended the ED out of hours spent longer in the ED (OR=1.329, 95% CI=1.315 to 1.344), were more likely to be admitted to hospital (OR=1.194, 95% CI=1.181 to 1.207) and were more likely to reattend (OR=1.067, 95% CI=1.054 to 1.081). The larger the ED (expressed as the number of attendances), the higher the likelihood of waiting more than 4 hours and of reattendance, but the lower the likelihood of hospital admission. The effect sizes associated with number of attendances per senior doctor were very close to OR=1 for all three outcomes.

### Analysis of ED performance

**Figure 3:**
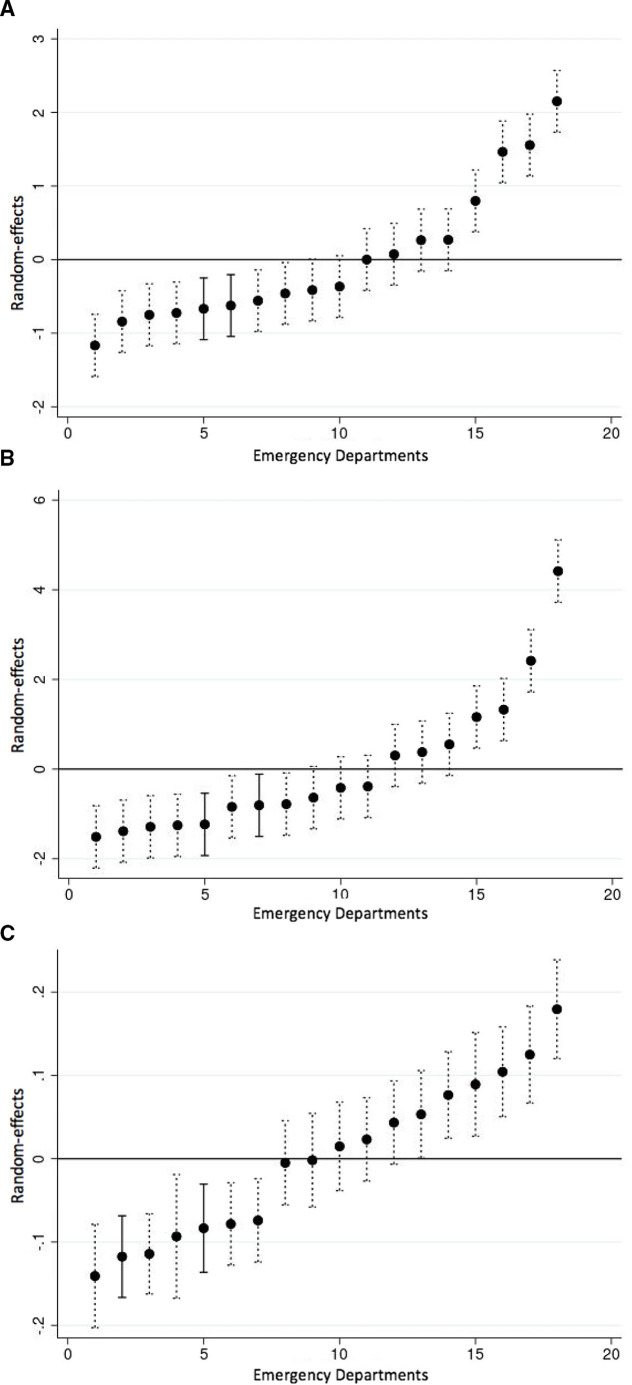
ED outcome random effects (18 sites) (2012–2017). (A) ED duration (>4 hours); (B) hospital admission from ED; (C) ED reattendance within 30 days.

The regression analyses allowed an assessment of the specific impact of the ED on patient outcomes, over and above the influence of patient and pathway characteristics, and their size and staffing ratios. Var(j) in table 2 indicates there is significant variation among the 18 type 1 EDs in the outcomes experienced by their patients. Figure 3 plots the ED random effects and 95% CIs, indicating each ED’s relative performance. Two EDs, indicated as solid lines, stood out as having significantly better performance than the average ED in Y&H with respect to all three outcomes. Patients in these two EDs were less likely to wait more than 4 hours, to be admitted to hospital or to reattend than those attending EDs elsewhere. This conclusion was robust to controlling for the bed occupancy rate in the host hospital trust (Table A2 and Figure A2 of the online supplemental material 1).

### Subgroup analyses

Table A3 in the appendix reports the average outcomes for the subgroups while Figure A3 and Table A4 show the regression results for all three outcomes, according to whether patients attended the ED either by ambulance or by other means. There are clear differences between these two groups in what factors are associated with their outcomes. For those who arrived by ambulance, few of the personal characteristics were particularly influential at explaining the likelihood of waiting more than 4 hours, other than out-of-hours attendance (OR=1.223, 95% CI=1.206 to 1.241). Out-of-hours attendance was also the most influential factor (OR=1.513, 95% CI=1.486 to 1.540) in explaining the probability of waiting more than 4 hours for those who did not arrive at the ED by ambulance, but there was also an age gradient, as older patients faced longer ED waits, as did those from care homes (OR=1.168, 95% CI=1.143 to 1.193).

For those arriving by ambulance, the most important factors associated with admission to hospital were the call handler designation of urgency: those considered to have urgent (OR=1.347, 95% CI=1.322 to 1.373), emergency (OR=1.379, 95% CI=1.360 to 1.399) or life-threatening conditions (OR 1.640, 95% CI=1.537 to 1.751) were more likely to be admitted than those designated less urgent. Older patients were also more likely to be admitted. Age was also an important predictor of admission for those who did not arrive by ambulance, and the likelihood of admission increases the more times they had attended the ED previously and if they attended out of hours (OR=1.48, 95% CI=1.457 to 1.503).

The factors associated with 30-day reattendance were little different for those who did and did not arrive at the ED by ambulance, previous attendances being the dominant factor.

The subgroup analysis of whether patients waited more than 4 hours in the ED for those who were and were not subsequently admitted to hospital is shown in Figure A4 and Table A5. Three sets of variables stand out as having a differential effect between these two groups. First, older age had a stronger association with how long patients waited among those who were not admitted compared with those admitted. Second, call handler designation was strongly associated with spending more than 4 hours in the ED for those who were not admitted. Third, out-of-hours attendance had a lesser association with long ED duration for those who were subsequently admitted than for those who were not (OR=1.170, 95% CI=1.154 to 1.187 and OR=1.562, 95% CI=1.532 to 1.593).

## Discussion

Our paper offers three key contributions to the literature examining ED performance. While there are many ways to measure ED performance,^13 14^ policymakers and the majority of empirical papers focus on just a single measure, typically time spent in the ED,^15–20^ it being known that ED delays are harmful.^7^ We follow the few studies that have considered multiple measures^21 22^ by analysing three ED outcomes, namely whether patients waited in ED for more than 4 hours, were admitted from ED to hospital and reattended the ED within 30 days of discharge.

Second, our analyses recognise that EDs do not operate in isolation. We were able to contextualise ED performance as part of the urgent care pathway, notably by accounting for calls to emergency services and use of ambulance services which emerged as important predictors of outcomes.

Third, we identified the impact that each specific ED had on performance. This involved controlling for a rich set of variables including patient characteristics, use of urgent care services prior to ED attendance, the time and day of attendance, and the size and staffing of the ED. Having accounted for the influence of these variables, we identified two EDs whose patients were significantly less likely than patients in other EDs to wait more than 4 hours, to be admitted to hospital and to reattend within 30 days. Qualitative study of one of these EDs highlighted the importance of a ‘frailty mindset’ in delivering high-quality, responsive services, including the development of pathways and roles specifically oriented towards the needs of frail older patients, and supporting positive risk-taking in considering options for those attending ED.^23^

Some of these factors associated with ED outcomes are already well known, notably that those of higher age, with a history of attendance, who attend out of hours and are from more deprived areas have a greater likelihood of a longer ED wait, hospital admission and ED reattendance.^24–28^ But we offer novel insights. First, our subgroup analysis revealed that personal characteristics were not strong influences on long waits for those who arrived by ambulance, suggesting they were afforded priority based primarily on their clinical urgency rather than any other characteristic. Second, we identified the importance of call handler designation for those conveyed to the ED by ambulance. This could be used to identify upon arrival at ED those at the highest risk of a long ED wait, hospital admission and ED reattendance. Call handler designations are routinely captured in electronic Patient Report Forms but often not in handwritten ones, and may not be routinely considered by professionals working in the ED. Given the characteristics of those prioritised urgent by call handlers, and the emerging evidence base for frailty-attuned interventions in the ED,^2 29^ it might be that senior decision-makers (qualified specialists, senior trainees or other senior healthcare staff such as advanced nurse practitioners) delivering initial assessment could use the designation to inform early intervention discussions or involvement of professionals with frailty expertise. Third, we identified that the ED itself may be a factor in explaining the three outcomes.

Our study demonstrates that performance can be measured not only with reference to a time-based standard but using a combination of metrics: ED duration (>4 hours), hospital admission from ED and ED reattendance within 30 days. This triple metric has the advantage of taking account of ED disposition, admission and reattendance, providing a more holistic, system-oriented service measure. As healthcare systems start to operate in more integrated ways (for example, Integrated Care Systems in England^30^), relatively simple barometers of system-wide urgent care pathway performance might be attractive to policymakers and commissioners. In combination with more person-centred metrics (for example, Patient-Reported Outcome Measures which are being developed for urgent care for older patients living with frailty^31 32^), the triple metric might help drive more person-centred, system-based emergency care approaches.

The strengths of this study include the large longitudinal dataset which captured information from a region with diverse EDs. We controlled for a wide range of patient characteristics and measures of the urgent care pathway not considered in previous studies of ED performance.

Our analysis was limited to routinely available data, which prioritise service and system-level over patient-centred outcomes.^33^ Even though diagnoses are likely to be important predictors of ED outcomes,^34^ unfortunately the ED data in the CUREd database do not capture diagnoses codes accurately or in a consistent fashion across EDs, with 61.5% of attendances having no diagnostic information recorded at all. While frailty identification is now commonplace in EDs,^35^ no measure of frailty could be constructed from our data for those attending the ED, only for those admitted to hospital.^36^ The staffing data identify only senior doctors but no other staff working in the ED and only quarterly data about bed occupancy rates were available for the period covered. It may be that the outcomes seen in the two ‘high-performing’ sites were related to the ED ‘model of care’ or wider organisational characteristics, such as an improvement culture.^23^

In summary, we have presented a comprehensive analysis of urgent care use by older patients to assess factors associated with the probabilities of a long ED wait, hospital admission and ED reattendance. While confirming the previously established influence of factors such as age, history of use and out-of-hours attendance, we identified that the emergency call handler designation was the most powerful predictor. The call handler designation could be added to other risk stratification measures (early warning scores, frailty measures) to identify patients with the highest risk of long ED waits, admission or reattendance, prompting assessment by a senior clinician at the point of arrival.

## Data Availability

Data may be obtained from a third party and are not publicly available. Retrospective, deidentified data were obtained from the CUREd Research Database (ref: CUREdRQ0004, CUREd data release register: https://docs.google.com/spreadsheets/d/1Fk1rZG9Y5AYIxuPj8sG4THNp671p8VpjYpLqa9BWxC8/edituspsharing) hosted by the CURE Group, University of Sheffield. Data were provided under a data sharing agreement which prohibits onward sharing. Other researchers may make their own requests for CUREd data via: https://www.sheffield.ac.uk/scharr/research/centres/cure/projects/cured-how-access-data.
