# Supplementary material for: Factors associated with longer wait times, admission and reattendances in older patients attending emergency departments: an analysis of linked healthcare data
Source: Emerg Med J. 2023 Jan 17;40(4):248–56. doi: 10.1136/emermed-2022-212303 (PMC10086302; doi:10.1136/emermed-2022-212303)
Supplement: Supplementary data [file emermed-2022-212303supp001.pdf]

Factors associated with longer wait times, admission and reattendances in older patients attending emergency departments. An analysis of linked healthcare data

Laia Maynou[1,2,3], Andrew Street[2], Chris Burton[4], Suzanne Mason[4], Tony Stone[4], Graham Martin[5], James van Oppen[6] and Simon Conroy[7]

[1]Department of Econometrics, Statistics and Applied Economics, Universitat de Barcelona, Avinguda Diagonal, 690, 08034, Barcelona, Spain

[2]Department of Health Policy, London School of Economics and Political Science, Houghton Street, London, WC2A 2AE, UK

[3]Center for Research in Health and Economics (CRES), Universitat Pompeu Fabra, Ramon Trias Fargas 25-27 08005 Barcelona, Spain

[4]CURE group, School of Health and Related Research, University of Sheffield, S1 4DA, UK

[5]THIS Institute, University of Cambridge, Clifford Allbutt Building, Cambridge Biomedical Campus, Cambridge, CB2 0AH, UK

[6]Department of Health Sciences, University of Leicester, George Davies Centre, University Road, Leicester, LE1 7RH, UK

[7]University College London, Medical Research Council Unit for Lifelong Health and Ageing at UCL, 5th Floor, 1-19 Torrington Place, London, WC1E 7HB, UK

Maynou L, *et al. Emerg Med J* 2023; 40:248–256. doi: 10.1136/emermed-2022-212303

Appendix

Description of data and variables

The analyses employed the CUREd research database, which links NHS data at individual level from NHS111, the Yorkshire Ambulance Service Computer Aided Dispatch (CAD) Data, the Emergency Department Patient Administration Systems (PAS) and the Admitted Patient Care (APC) dataset. The linkage is described in Lewis et al [1]. The analytical sample comprised all those older aged 75 or older who attended a type 1 ED in the Yorkshire and Humber region between April 2012 and March 2017.

We analysed three outcomes for patients attending ED. The first outcome was whether they were seen and discharged from the ED within four hours of arrival, calculated by comparing time of arrival in the ED and departure time from the ED. The second was whether they were transferred from the ED and admitted to hospital, determined by seeing if the same patient appeared in the APC dataset within 24 hours of being transferred from the ED. A 24 hour window was applied because only dates not time is recorded in the APC, and some people are transferred overnight. The third was whether the patient re-attended the ED within 30 days of discharge either from the ED or hospital, this being ascertained by looking at the gap between dates of ED discharge and subsequent attendance in the linked data.

The analyses controlled for a set of socio-demographic and clinical characteristics including: the patients' age, categorized into 5-year age bands; sex; the socioeconomic conditions of where they lived using the deciles of Index of Multiple Deprivation (IMD) [2], with IMD=1 indicating the worst-off communities; number of ED attendances in the past year; whether the patient's postcode indicated that they were a care home resident; and the travel time by road between the patient´s residence and the hospital [3]. The analysis of re-attendance was conditioned on the patient surviving the ED or hospital admission, identified from the PAS attendance disposal and APC discharge method variables.

We included a set of variables capturing the patient's emergency and urgent care journey prior to admission. For those that made emergency calls, we accounted for the number and length in minutes of the individual´s emergency (NHS111 and 999) calls. For those conveyed to the ED ambulance, we included variables measuring: the time of the ambulance on scene (arrival to departure); the time taken between calling the ambulance and arrival at the emergency department; and the urgency with which the ambulance was dispatched, assigned by the NHS Pathways triage system based on answers from the caller to scripted questions asked by the call-handler [4].

We included variables accounting for the day of ED attendance, whether or not the ED attendance was out-of-hours (all weekend and weekday from 6.30pm to 8am) and whether this was on a public holiday [5]; and month and financial year variables capturing seasonal effects and annual trends. These variables are not reported in the tables of results or forest plots.

Maynou L, *et al. Emerg Med J* 2023; 40:248–256. doi: 10.1136/emermed-2022-212303

We use number of attendances (in 1000s) during the year that the patient attended in order account for the size of the ED. We also construct patient-to-doctor ratios, using annual data constructed from NHS workforce statistics which record the number of full time equivalent senior A&E doctors (referred to as "consultants" in England) in each hospital trust [6]. For trusts with more than one A&E site, doctors were allocated according to the share of attendances.

Table A1: Multilevel model - ED duration, Hospital admission from ED and ED reattendance within 30 days - 2012-2017. Time variables

Dep var:	(1) ED duration (>4 hours)	(2) Hospital admission from ED	(3) 30-day reattendance
	Logit - Odds ratio	Logit - Odds ratio	Logit - Odds ratio
Bank holiday	0.979	1.116***	1.027*
	(0.953-1.007)	(1.086-1.147)	(0.996-1.060)
Thursday	0.933***	0.988	0.996
	(0.917-0.950)	(0.971-1.004)	(0.977-1.015)
Friday	0.899***	0.976***	0.996
	(0.883-0.914)	(0.960-0.993)	(0.978-1.015)
Saturday	0.794***	0.846***	0.971***
	(0.780-0.809)	(0.831-0.861)	(0.951-0.991)
Sunday	0.910***	0.874***	0.965***
	(0.894-0.927)	(0.858-0.890)	(0.945-0.984)
Monday	1.272***	0.971***	0.987
	(1.251-1.293)	(0.955-0.988)	(0.968-1.005)
Tuesday	1.106***	0.985*	0.967***
	(1.088-1.125)	(0.969-1.002)	(0.948-0.985)
May	0.845***	0.928***	0.996
	(0.826-0.864)	(0.908-0.948)	(0.972-1.021)
June	0.874***	0.922***	0.978*
	(0.854-0.895)	(0.901-0.942)	(0.954-1.003)
July	0.859***	0.909***	0.970**
	(0.839-0.878)	(0.890-0.930)	(0.946-0.994)
August	0.865***	0.914***	0.992
	(0.0.845-0.885)	(0.894-0.934)	(0.967-1.016)
September	0.955***	0.940***	0.968**
	(0.933-0.977)	(0.920-0.961)	(0.944-0.993)
October	1.183***	0.970***	0.978*
	(1.157-1.209)	(0.949-0.992)	(0.954-1.002)
November	1.238***	0.988	1.002
	(1.211-1.266)	(0.967-1.011)	(0.978-1.028)
December	1.403***	0.978**	1.015
	(1.373-1.434)	(0.958-0.999)	(0.991-1.040)
January	1.444***	1.003	0.982
	(1.413-1.476)	(0.982-1.025)	(0.958-1.006)
February	1.390***	0.991	0.975*
	(1.360-1.422)	(0.969-1.014)	(0.951-1.0002)
March	1.204***	0.942***	0.857***
	(1.178-1.231)	(0.922-0.963)	(0.836-0.879)
2013-2014	0.911***	1.088***	1.006
	(0.896-0.927)	(1.071-1.105)	(0.988-1.024)
2014-2015	1.241***	1.342***	0.986
	(1.220-1.261)	(1.321-1.363)	(0.968-1.004)
2015-2016	1.320***	1.437***	0.995
	(1.297-1.344)	(1.412-1.462)	(0.977-1.014)
2016-2017	1.424***	1.588***	0.953***
	(1.393-1.455)	(1.555-1.622)	(0.931-0.975)
N	990,172	990,645	990,229
Sites	18	18	18
Time Fixed-Effects (day, month, financial year)	Yes	Yes	Yes
Random-Effects levels	Site & patient	Site & patient	Site & patient
Years	2012-2017	2012-2017	2012-2017

*Notes: See note to Table 1 for abbreviations. Significance levels: *** p<0.01, ** p<0.05, * p<0.1. Odds ratios reported and 95% confidence interval (CI) in parentheses. Model 3 (30-day reattendance) includes death as a covariate. Reference categories: No bank holiday, Wednesday, April and 2012-2013.*

Figure A1: ED outcomes estimates (2012-2017) all variables

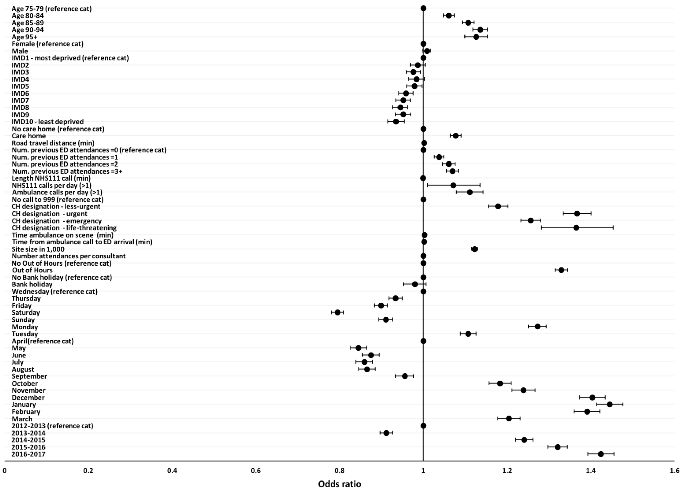

(I) - ED duration (>4 hours)

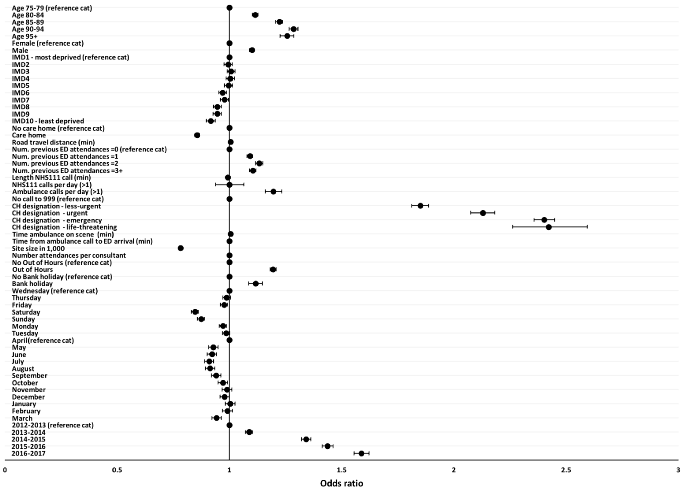

(II) - Hospital admission from ED

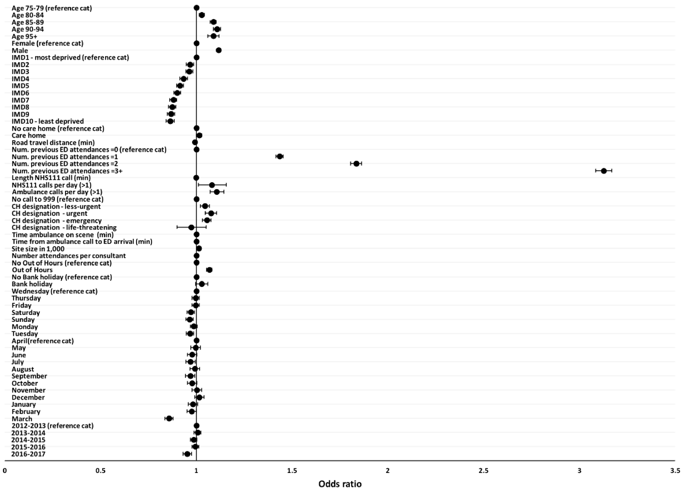

(III) - ED re-attendance within 30 days

Notes: See note to Table 1 for abbreviations.

Robustness check: accounting for bed occupancy rate

There is evidence that ED performance is related to capacity constraints, notably the availability of beds, in the host hospital [7; 8; 9]. To explore this, we ran analyses that also controlled for the percentage of overnight hospital beds that are occupied using quarterly data reported by NHS England for each hospital Trust [10]. These quarterly data are an imperfect indication of whether or not a bed might be available for any particular person requiring admission from the ED but daily data were unavailable for the period covered by our study.

When including quarterly bed occupancy rate, the model estimating the probability of hospital admission failed to converge unless the year dummies were omitted. Omitting year dummies biases the estimates of most of the other variables in the model away from OR=1, whereas omission of the bed occupancy rate has negligible impact on the other estimates. Hence, the decision was made to retain year dummies and omit bed occupancy rate in the main analysis.

Conclusions about the relative performance of EDs were also robust to omission or inclusion of the bed occupancy rate variable. The same two ED sites were identified as having patients that were significantly less likely than patients in other EDs to wait more than four hours, to be admitted to hospital and to re-attend within 30 days. Similarly, no other ED was found in which patients were significantly less (or more) likely than the national average to have longer waits, be admitted or re-attend when accounting for bed occupancy rate.

Maynou L, *et al. Emerg Med J* 2023; 40:248–256. doi: 10.1136/emermed-2022-212303

Table A2: Multilevel model - ED duration, Hospital admission from ED and ED reattendance within 30 days - 2012-2017. Robustness check (occupancy rate)

Dep var:	(1) ED duration (>4 hours)	(2) Hosp admission from ED	(3) 30-day reattedance
	Logit - Odds ratio	Logit - Odds ratio	Logit - Odds ratio
Age 80-85	1.061***	1.128***	1.028***
	(1.048-1.074)	(1.116-1.141)	(1.015-1.042)
Age 85-90	1.107***	1.255***	1.088***
	(1.093-1.121)	(1.240-1.269)	(1.073-1.104)
Age 90-95	1.135***	1.332***	1.107***
	(1.118-1.153)	(1.313-1.351)	(1.089-1.126)
Age 95+	1.126***	1.300***	1.089***
	(1.099-1.154)	(1.270-1.331)	(1.059-1.119)
Sex (=1 male)	1.008*	1.118***	1.116***
	(0.999-1.017)	(1.108-1.128)	(1.105-1.128)
IMD2	0.987	0.994	0.967***
	(0.969-1.005)	(0.977-1.011)	(0.949-0.986)
IMD3	0.974***	1.009	0.963***
	(0.957-0.991)	(0.993-1.026)	(0.945-0.982)
IMD4	0.983*	1.005	0.934***
	(0.964-1.002)	(0.987-1.023)	(0.914-0.954)
IMD5	0.978**	0.995	0.915***
	(0.959-0.996)	(0.977-1.012)	(0.896-0.934)
IMD6	0.958***	0.968***	0.900***
	(0.941-0.976)	(0.951-0.984)	(0.882-0.919)
IMD7	0.952***	0.976***	0.880***
	(0.935-0.969)	(0.959-0.993)	(0.862-0.898)
IMD8	0.945***	0.941***	0.874***
	(0.927-0.963)	(0.925-0.958)	(0.856-0.893)
IMD9	0.950***	0.937***	0.868***
	(0.932-0.969)	(0.921-0.955)	(0.849-0.887)
IMD10 - least deprived	0.934***	0.905***	0.863***
	(0.914-0.954)	(0.887-0.923)	(0.842-0.884)
Care Home (=1 yes)	1.075***	0.832***	1.014**
	(1.062-1.088)	(0.822-0.842)	(1.0001-1.028)
Road travel distance (min)	1.001***	1.006***	0.993***
	(1.001-1.002)	(1.005-1.006)	(0.992-0.994)
Num. previous ED attendances =1	1.037***	1.110***	1.434***
	(1.025-1.049)	(1.099-1.122)	(1.415-1.453)
Num. previous ED attendances =2	1.061***	1.162***	1.834***
	(1.046-1.077)	(1.146-1.177)	(1.806-1.863)
Num. previous ED attendances =3+	1.071***	1.131***	3.126***
	(1.058-1.085)	(1.117-1.145)	(3.085-3.168)
Length NHS111 call (min)	0.999***	0.993***	0.999***
	(0.998-0.999)	(0.992-0.993)	(0.998-0.999)
NHS111 calls per day (>1)	1.078**	1.002	1.081**
	(1.016-1.143)	(0.945-1.064)	(1.012-1.155)
Ambulance calls per day (>1)	1.112***	1.280***	1.107***
	(1.081-1.145)	(1.243-1.318)	(1.071-1.143)
CH designation - less-urgent	1.173***	1.828***	1.045***
	(1.150-1.197)	(1.794-1.864)	(1.022-1.069)
CH designation - urgent	1.359***	2.427***	1.076***
	(1.326-1.392)	(2.372-2.482)	(1.046-1.107)
CH designation - emergency	1.254***	2.529***	1.054***
	(1.231-1.278)	(2.484-2.576)	(1.032-1.077)
CH designation - life-threatening	1.375***	2.837***	0.972
	(1.290-1.465)	(2.656-3.030)	(0.899-1.052)
Time ambulance on scene (min)	1.003***	1.007***	1.001**
	(1.002-1.003)	(1.007-1.008)	(1.0001-1.001)
Time from ambulance call to ED arrival (min)	1.002***	1.0001	1.0004***
	(1.001-1.002)	(0.999-1.0002)	(1.0002-1.001)
Out of Hours (=1 yes)	1.330***	1.227***	1.067***
	(1.315-1.344)	(1.214-1.240)	(1.054-1.081)
Site size in 1,000	1.139***	0.865***	1.013***
	(1.132-1.146)	(0.861-0.869)	(1.007-1.019)
Number attendances per consultant	0.999**	1.00002**	1.00002*
	(0.999-0.999)	(1.000001-1.00004)	(0.999-1.00004)
Occupancy rate per trust	1.028***	1.003***	0.999*
	(1.027-1.030)	(1.002-1.004)	(0.997-1.0001)
Var(j)	0.821***	0.605***	0.008***
	(0.284-1.357)	(0.210-1.0001)	(0.003-0.014)
N	990,172	990,645	990,229
Sites	18	18	18
Time Fixed-Effects (day, month, financial year)	Yes	Yes+	Yes
Random-Effects levels	Site & patient	Site & patient	Site & patient
Years	2012-2017	2012-2017	2012-2017

*Notes: See note to Table 1 for abbreviations. Significance levels: *** p<0.01, ** p<0.05, * p<0.1. Odds ratios reported and 95% CI in parentheses. Var(j) reports the estimate and the 95% CI in parentheses. Model 3 (30-day reattendance) includes death as a covariate. Reference categories: Age 75-79, Female, IMD1 - most deprived, Previous ED attendances=0, No care home, NHS111 calls per <1, Ambulance (999) per day <1, No call to 999, No Out of Hours.+financial year dummies not included.*

Maynou L, et al. Emerg Med J 2023; 40:248–256. doi: 10.1136/emermed-2022-212303

Figure A2: ED outcomes residuals (18 sites) - (2012-2017) - Robustness check (with occupancy rate).

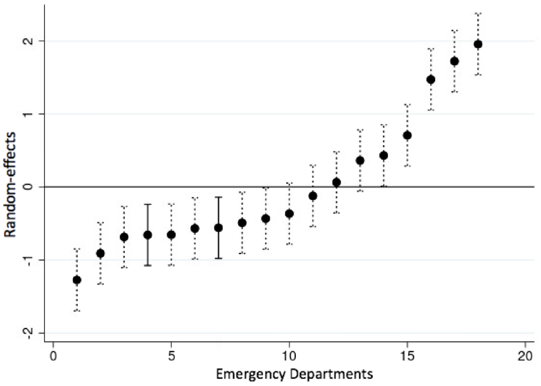

(I) - ED duration (>4 hours)

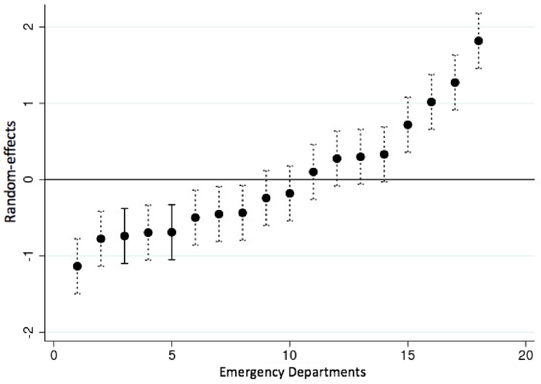

(II) - Hospital admission from ED

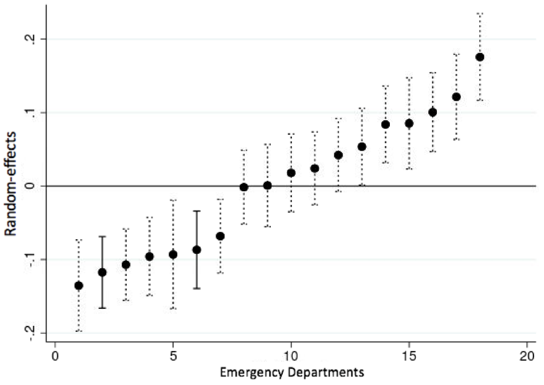

(III) - ED reattendance within 30 days

Maynou L, *et al. Emerg Med J* 2023; 40:248–256. doi: 10.1136/emermed-2022-212303

Sub-group analyses

Table A3: Outcomes by sub-groups

	**Ambulance** N=552,940	**Non-Ambulance** N=486,311	**Admitted to hosp.** N=600,368	**Non-admitted to hosp.** N=438,883
**ED duration (>4 hours)**	33.43 (47.18)	22.29 (41.62)	34.71 (47.61)	19.32 (39.48)
**Hospital admission from ED**	70.71 (45.51)	43.06 (49.52)		
**ED reattendance within 30 days**	21.66 (41.19)	19.00 (39.23)		

Notes: See note to Table 1 for abbreviations. % (SD)

Table A4: Multilevel model - ED outcomes' results sub-group analysis (Ambulance vs Non-ambulance): ED duration, Hospital admission from ED and ED reattendance within 30 days - 2012-2017.

Dep var:	(1) ED duration (>4 hours)	(2)	(3) Hosp. adm. from ED	(4)	(5) 30-days reattendance	(6)
	**Ambulance**	**Non-Amb**	**Ambulance**	**Non-Amb**	**Ambulance**	**Non-Amb**
**Age 80-85**	1.010	1.111***	1.059***	1.169***	1.015	1.038***
	(0.994-1.027)	(1.092-1.131)	(1.042-1.076)	(1.151-1.187)	(0.996-1.034)	(1.018-1.058)
**Age 85-90**	1.017**	1.219***	1.109***	1.371***	1.056***	1.122***
	(1.0003-1.035)	(1.196-1.243)	(1.090-1.127)	(1.348-1.394)	(1.036-1.077)	(1.099-1.146)
**Age 90-95**	1.029***	1.290***	1.142***	1.517***	1.073***	1.147***
	(1.009-1.049)	(1.258-1.322)	(1.120-1.165)	(1.485-1.550)	(1.049-1.097)	(1.117-1.178)
**Age 95+**	1.001	1.329***	1.084***	1.551***	1.063***	1.115***
	(0.971-1.032)	(1.275-1.386)	(1.052-1.117)	(1.493-1.610)	(1.027-1.101)	(1.065-1.167)
**Sex (=1 male)**	0.999	1.018**	1.091***	1.143***	1.110***	1.123***
	(0.987-1.011)	(1.004-1.033)	(1.077-1.104)	(1.129-1.157)	(1.095-1.126)	(1.106-1.140)
**IMD2**	0.989	0.990	0.998	0.997	0.976*	0.957***
	(0.966-1.012)	(0.962-1.018)	(0.976-1.022)	(0.973-1.023)	(0.951-1.002)	(0.929-0.987)
**IMD3**	0.978*	0.976*	1.019	1.004	0.975*	0.948***
	(0.956-1.0003)	(0.949-1.003)	(0.996-1.042)	(0.980-1.028)	(0.951-1.001)	(0.921-0.977)
**IMD4**	1.003	0.963**	1.023*	0.992	0.958***	0.905***
	(0.979-1.028)	(0.935-0.993)	(0.998-1.048)	(0.966-1.019)	(0.932-0.985)	(0.876-0.935)
**IMD5**	0.979*	0.980	1.007	0.973**	0.919***	0.910***
	(0.955-1.003)	(0.952-1.009)	(0.983-1.032)	(0.949-0.998)	(0.893-0.945)	(0.883-0.939)
**IMD6**	0.979*	0.941***	1.032***	0.910***	0.889***	0.914***
	(0.956-1.002)	(0.915-0.968)	(1.008-1.057)	(0.888-0.933)	(0.865-0.914)	(0.887-0.942)
**IMD7**	0.966***	0.949***	1.032***	0.934***	0.884***	0.878***
	(0.943-0.989)	(0.922-0.976)	(1.008-1.057)	(0.911-0.957)	(0.860-0.908)	(0.852-0.906)
**IMD8**	0.964***	0.930***	0.996	0.900***	0.863***	0.888***
	(0.940-0.989)	(0.903-0.958)	(0.971-1.021)	(0.877-0.923)	(0.838-0.888)	(0.861-0.917)
**IMD9**	0.981	0.932***	0.997	0.893***	0.870***	0.868***
	(0.956-1.007)	(0.905-0.960)	(0.971-1.023)	(0.870-0.916)	(0.844-0.896)	(0.840-0.896)
**IMD10 - least deprived**	0.964***	0.906***	0.973*	0.858***	0.845***	0.880***
	(0.937-0.991)	(0.876-0.937)	(0.947-1.001)	(0.833-0.884)	(0.818-0.873)	(0.849-0.914)
**Care Home (=1 yes)**	1.029***	1.168***	0.716***	1.067***	0.989	1.059***
	(1.014-1.045)	(1.143-1.193)	(0.705-0.727)	(1.047-1.087)	(0.973-1.006)	(1.035-1.083)
**Road travel distance (min)**	0.998***	1.004***	1.001***	1.008***	0.994***	0.992***
	(0.997-0.999)	(1.003-1.005)	(1.001-1.002)	(1.008-1.009)	(0.993-0.995)	(0.992-0.993)
**Num. previous ED att. =1**	1.034***	1.038***	1.069***	1.137***	1.388***	1.476***
	(1.018-1.050)	(1.019-1.056)	(1.053-1.085)	(1.120-1.154)	(1.363-1.414)	(1.448-1.505)
**Num. previous ED att. =2**	1.051***	1.067***	1.079***	1.234***	1.768***	1.899***
	(1.032-1.070)	(1.042-1.091)	(1.060-1.100)	(1.210-1.259)	(1.731-1.805)	(1.855-1.945)
**Num. previous ED att. =3+**	1.025***	1.143***	0.988	1.326***	3.010***	3.248***
	(1.008-1.042)	(1.119-1.168)	(0.972-1.004)	(1.302-1.351)	(2.957-3.064)	(3.181-3.315)
**Length NHS111 call (min)**	0.999	0.992***	0.994***	0.979***	0.999	0.992***
	(0.998-1.003)	(0.989-0.994)	(0.994-0.995)	(0.977-0.981)	(0.999-1.001)	(0.989-0.994)
**NHS111 calls per day (>1)**	1.078**	0.694**	0.977	0.898	1.084**	0.918
	(1.015-1.145)	(0.519-0.926)	(0.918-1.039)	(0.703-1.147)	(1.012-1.161)	(0.681-1.238)
**Ambulance calls per day (>1)**	1.100***		1.311***		1.101***	
	(1.069-1.132)		(1.273-1.351)		(1.065-1.137)	
**CH desig. - urgent**	1.059***		1.347***		1.008	
	(1.029-1.090)		(1.322-1.373)		(0.975-1.042)	
**CH desig. - emergency**	1.058***		1.379***		1.004	
	(1.042-1.073)		(1.360-1.399)		(0.988-1.021)	
**CH desig. - life-threatening**	1.076**		1.640***		0.913**	
	(1.008-1.148)		(1.537-1.751)		(0.843-0.990)	
**Time ambulance on scene (min)**	1.003***		1.008***		1.001***	
	(1.002-1.003)		(1.008-1.008)		(1.0002-1.001)	
**Time from ambulance call to ED arrival (min)**	1.001***		1.001***		1.0004***	
	(1.001-1.002)		(1.0003-1.001)		(1.0002-1.001)	
**Out of Hours (=1 yes)**	1.223***	1.513***	1.039***	1.480***	1.038***	1.112***
	(1.206-1.241)	(1.486-1.540)	(1.024-1.054)	(1.457-1.503)	(1.021-1.055)	(1.090-1.133)
**Site size in 1,000**	1.138***	1.079***	0.857***	0.877***	1.012***	1.009**
	(1.130-1.147)	(1.067-1.091)	(0.852-0.862)	(0.871-0.882)	(1.006-1.019)	(1.0002-1.018)
**Number att. per cons.**	0.999	0.999***	0.999***	1.0001***	1.00004**	1.00002
	(0.999-1.00002)	(0.999-0.999)	(0.999-0.999)	(1.00003-1.0001)	(1.00001-1.0001)	(0.999-1.0001)
**Var(j)**	0.819***	0.803***	0.637***	0.701***	0.006***	0.012***
	(0.227-1.361)	(0.280-1.327)	(0.216-1.057)	(0.240-1.163)	(0.002-0.011)	(0.004-0.019)
**N**	523,247	466,925	523,506	467,139	523,277	466,952
**Sites**	18	18	18	18	18	18
**Time Fixed-Effects**	Yes	Yes	Yes^{+}	Yes^{+}	Yes	Yes
**Random-Effects levels**	Site & patient	Site & patient	Site & patient	Site & patient	Site & patient	Site & patient
**Years**	2012-2017	2012-2017	2012-2017	2012-2017	2012-2017	2012-2017

*Notes: See note to Table 1 for abbreviations. Significance levels: *** p<0.01, ** p<0.05, * p<0.1. Odds ratios reported and 95% CI in parentheses. Var(j) reports the estimate and the 95% CI in parentheses. Model 5 and 6 (30-day re-attendance) include death as a covariate. Reference categories: Age 75-79, Female, IMD1 - most deprived, Previous ED attendances=0, No care home, NHS111 calls per <1, Ambulance (999) per day <1, CH designation - less-urgent, No Out of Hours. $^{+}$financial year dummies not included.*

Maynou L, *et al. Emerg Med J* 2023; 40:248–256. doi: 10.1136/emermed-2022-212303

Table A5: Multilevel model - ED outcomes' results sub-group analysis (Admitted to hospital vs Non-admitted to hospital): ED duration. - 2012-2017.

	(1)	(2)
**Dep var:**	ED duration (>4 hours)	
	**Admitted to hospital**	**Non-admitted to hospital**
**Age 80-85**	1.010<br>(0.995-1.016)	1.077***<br>(1.055-1.099)
**Age 85-90**	1.010<br>(0.993-1.026)	1.153***<br>(1.128-1.179)
**Age 90-95**	1.014<br>(0.995-1.033)	1.200***<br>(1.168-1.232)
**Age 95+**	0.976<br>(0.946-1.006)	1.276***<br>(1.223-1.331)
**Sex (=1 male)**	0.979***<br>(0.967-0.991)	1.004<br>(0.988-1.021)
**IMD2**	0.978*<br>(0.956-1.001)	1.003<br>(0.973-1.035)
**IMD3**	0.969***<br>(0.948-0.991)	0.993<br>(0.963-1.024)
**IMD4**	0.989<br>(0.965-1.013)	0.990<br>(0.958-1.023)
**IMD5**	0.981<br>(0.958-1.004)	0.977<br>(0.946-1.010)
**IMD6**	0.970***<br>(0.948-0.993)	0.968**<br>(0.939-0.999)
**IMD7**	0.963***<br>(0.941-0.986)	0.962**<br>(0.932-0.993)
**IMD8**	0.965***<br>(0.941-0.989)	0.951***<br>(0.920-0.983)
**IMD9**	0.964***<br>(0.941-0.989)	0.970*<br>(0.938-1.004)
**IMD10 - least deprived**	0.962***<br>(0.936-0.989)	0.930***<br>(0.896-0.965)
**Care Home (=1 yes)**	1.059***<br>(1.043-1.076)	1.209***<br>(1.183-1.235)
**Road travel distance (min)**	0.998***<br>(0.997-0.999)	1.003***<br>(1.002-1.004)
**Num. previous ED attendances =1**	1.010<br>(0.995-1.025)	1.041***<br>(1.020-1.062)
**Num. previous ED attendances =2**	1.012<br>(0.994-1.031)	1.080***<br>(1.053-1.108)
**Num. previous ED attendances =3+**	1.004<br>(0.988-1.021)	1.137***<br>(1.112-1.163)
**Length NHS111 call (min)**	1.001***<br>(1.0003-1.002)	0.999**<br>(0.997-0.999)
**NHS111 calls per day (>1)**	1.152***<br>(1.072-1.237)	0.889**<br>(0.796-0.992)
**Ambulance calls per day (>1)**	1.063***<br>(1.027-1.099)	1.121***<br>(1.058-1.188)
**CH designation - less-urgent**	0.882***<br>(0.860-0.905)	1.311***<br>(1.266-1.358)
**CH designation - urgent**	0.923***<br>(0.894-0.952)	1.610***<br>(1.542-1.682)
**CH designation - emergency**	0.896***<br>(0.876-0.917)	1.436***<br>(1.387-1.487)
**CH designation - life-threatening**	0.890***<br>(0.826-0.959)	1.642***<br>(1.444-1.868)
**Time ambulance on scene (min)**	1.002***<br>(1.001-1.002)	1.002***<br>(1.002-1.003)
**Time from ambulance call to ED arrival (min)**	1.002***<br>(1.001-1.002)	1.002***<br>(1.001-1.002)
**Out of Hours (=1 yes)**	1.170***<br>(1.154-1.187)	1.562***<br>(1.532-1.593)
**Site size in 1,000**	1.182***<br>(0.1.173-1.191)	1.045***<br>(1.024-1.066)
**Number attendances per consultant**	1.0001***<br>(1.0001-1.0001)	0.999***<br>(0.999-0.999)
**Var(j)**	0.870***<br>(0.301-1.439)	1.063***<br>(0.368-1.758)
**N**	571,897	418,275
**Sites**	18	18
**Time Fixed-Effects (day, month, financial year)**	Yes	Yes
**Random-Effects levels**	Site & patient	Site & patient
**Years**	2012-2017	2012-2017

*Notes: See note to Table 1 for abbreviations. Significance levels: *** p<0.01, ** p<0.05, * p<0.1. Odds ratios reported and 95% CI in parentheses. Var(j) reports the estimate and the 95% CI in parentheses. Reference categories: Age 75-79, Female, IMD1 - most deprived, Previous ED attendances=0, No care home, NHS111 calls per <1, Ambulance (999) per day <1, No call to 999, No Out of Hours.*

Figure A3: ED outcomes estimates ambulance vs no ambulance - (2012-2017)

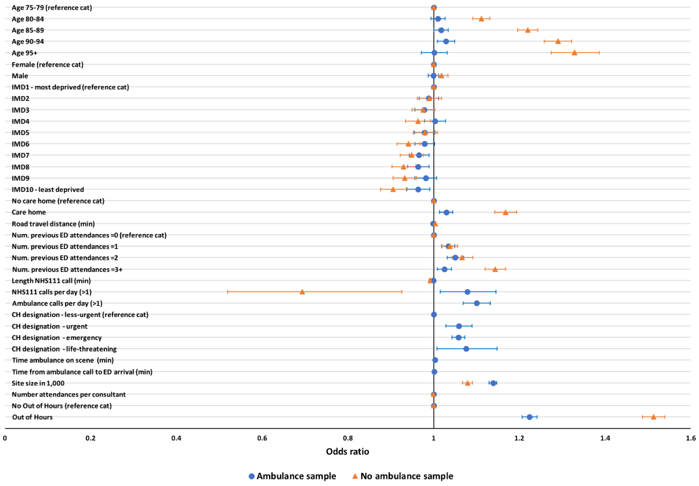

(I) - ED duration (>4 hours)

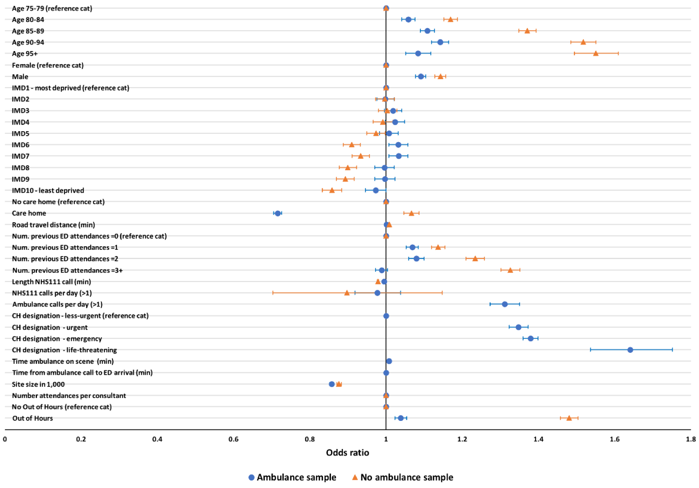

(II) - Hospital admission from ED

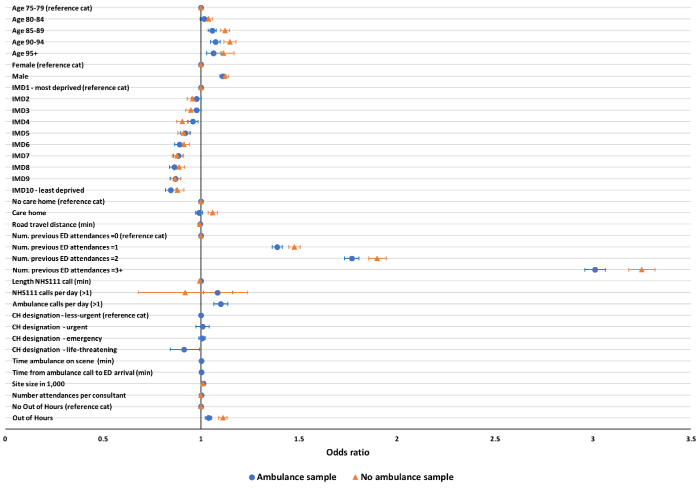

(III) - ED reattendance within 30 days

Notes: See note to Table 1 for abbreviations.

Figure A4: ED duration (>4 hours). Admitted to hospital vs Non-admitted to hospital

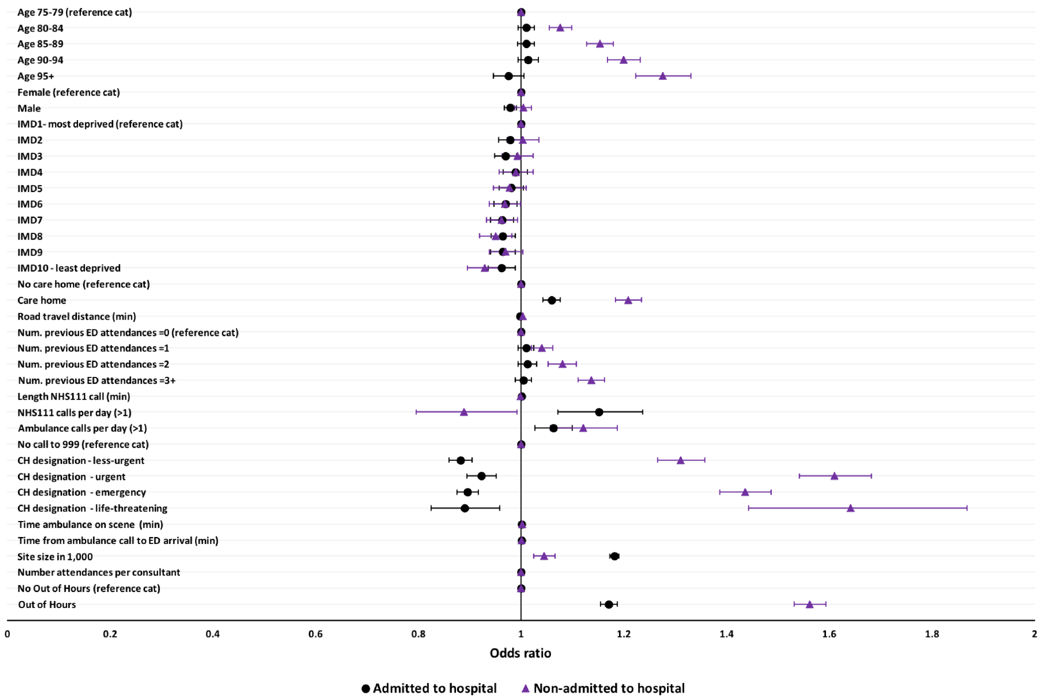

Notes: See note to Table 1 for abbreviations.

Maynou L, *et al. Emerg Med J* 2023; 40:248–256. doi: 10.1136/emermed-2022-212303

References

[1] Lewis J, Stone T, Simpson R, Jacques R, O'Keeffe C, Croft S, et al. Patient compliance with NHS 111 advice: Analysis of adult call and ED attendance data 2013–2017. Plos ONE. 2021;16(5):e0251362.

[2] Ministry of Housing, Communities and Local Government. The English indices of deprivation 2015;. Accessed June 12, 2020 [Online]. Available from: https://www.gov.uk/government/statistics/english-indices-of-deprivation-2015.

[3] Bankart MJ, Baker R, Rashid A, Habiba H, Banerjee J, Hsu R, et al. Characteristics of general practices associated with emergency admission rates to hospital: a cross-sectional study. Emergency Medicine Journal. 2011;28(7):558-63.

[4] Yorkshire Ambulance Service NHS Trust. What happens when you call 999?;. Accessed Apr. 19, 2022 [Online]. Available from: https://www.yas.nhs.uk/our-services/emergency-ambulance-service-999/what-happens-when-you-call-999/.

[5] Walker AS, Mason A, Quan TP, Fawcett NJ, Watkinson P, Llewelyn M, et al. Mortality risks associated with emergency admissions during weekends and public holidays: an analysis of electronic health records. The Lancet. 2017;390(10089):62-72.

[6] NHS Digital. NHS workforce statistics;. Accessed Apr. 19, 2022 [Online]. Available from: https://digital.nhs.uk/data-and-information/publications/statistical/nhs-workforce-statistics.

[7] Keogh B, Culliford D, Guerrero-Ludueña R, Monks T. Exploring emergency department 4-hour target performance and cancelled elective operations: a regression analysis of routinely collected and openly reported NHS trust data. BMJ Open. 2018;8(5).

[8] Friebel R, Juarez RM. Spill Over Effects of Inpatient Bed Capacity on Accident and Emergency Performance in England. Health Policy. 2020;124(11):1182-91.

[9] Gaughan J, Kasteridis P, Mason A, Street A. Why are there long waits at English emergency departments? The European Journal of Health Economics. 2020;21(2):209-18.

[10] NHS England. Bed availability and occupancy;. Accessed Apr. 19, 2022 [Online]. Available from: https://www.england.nhs.uk/statistics/statistical-work-areas/bed-availability-and-occupancy.

Maynou L, *et al. Emerg Med J* 2023; 40:248–256. doi: 10.1136/emermed-2022-212303
